# Supplementary material for: Capsaicin-Loaded Chitosan Nanocapsules for wtCFTR-mRNA Delivery to a Cystic Fibrosis Cell Line
Source: Biomedicines. 2020 Sep 20;8(9):364. doi: 10.3390/biomedicines8090364 (PMC7554911; doi:10.3390/biomedicines8090364)
Supplement: Supplementary file 1 [file biomedicines-08-00364-s001.pdf]

biomedicines

MDPI

Supplementary material

Capsaicin-loaded chitosan nanocapsules for wtCFTR-mRNA delivery to a cystic fibrosis cell line

A. Katharina Kolonko[1,*], Janes Efing[1], Yadira González-Espinosa[2], Nadine Bangel-Ruland[1], Willy van Driessche[3], Francisco M. Goycoolea[2], Wolf-Michael Weber[1]

1 Institute of Animal Physiology, University of Muenster, Schlossplatz 8, 48143 Muenster, Germany; j_efin04@wwu.de (J.E.); n.br@wwu.de (N.B.-R.); wmw@wwu.de (W.-M.W.)
2 School of Food Science and Nutrition, University of Leeds, Leeds, LS2 9JT, United Kingdom; Y.GonzalezEspinosa@leeds.ac.uk (Y.G.-E.); F.M.Goycoolea@leeds.ac.uk (F.M.G.)
3 EP-Devices, Tervuursesteenweg 154, 3060 Bertem, Belgium; ep.devices@telenet.be
* Correspondence:: katharina.kolonko@wwu.de; Tel.: +49-251-8321784

Table S1. Physicochemical properties of chitosan (CS) nanocapsules and CS nanocapsules surface-loaded with wtCFTR-mRNA at P/N charge ratio 75. (NC Blank) Blank nanocapsules; (NC CAP) nanocapsules loaded with capsaicin (*n*=3).

Attribute	NC Blank[1]	NC Blank-mRNA[2]	NC CAP[3]	NC CAP -mRNA[4]
Hydrodynamic diameter [nm]	137.5 ± 8.2	141.2 ± 5.9	216.6 ± 35.3	204.3 ± 10.2
Polydispersity index	0.18 ± 0.03	0.14 ± 0.01	0.11 ± 0.02	0.11 ± 0.02
Zeta potential [mV]	+58.3 ± 4.6	+57.2 ± 1.7	+61.3 ± 3.1	+63.1 ± 1.6

[1] Blank nanocapsules; [2] blank nanocapsules loaded with wtCFTR-mRNA; [3] capsaicin loaded nanocapsules; [4] capsaicin loaded nanocapsules co-loaded with wtCFTR-mRNA

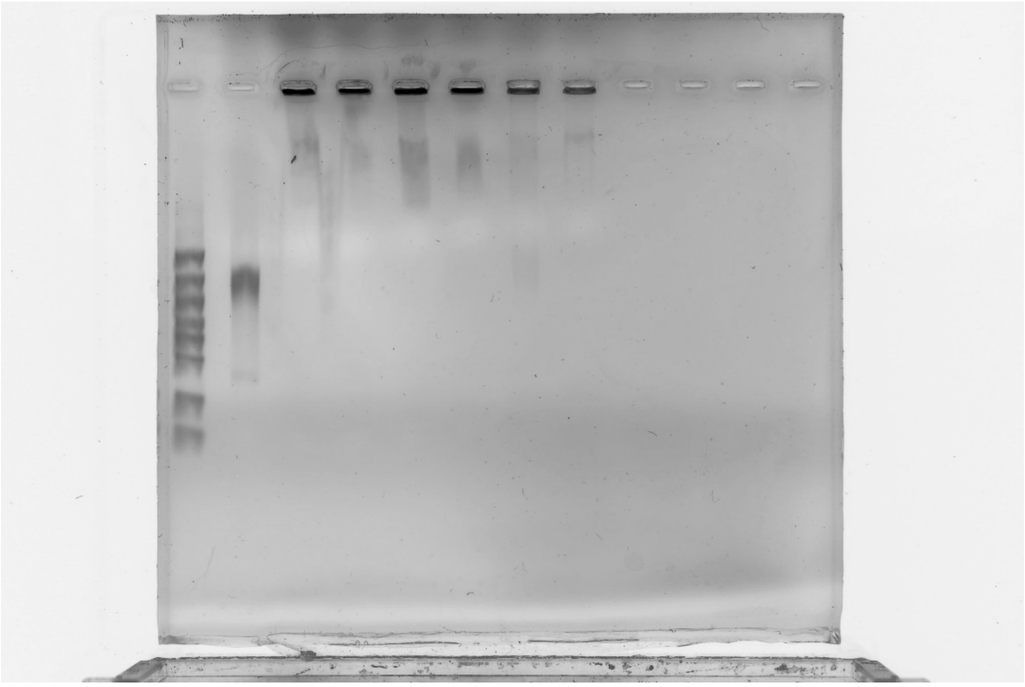

Figure S1. Original image of the gel retardation assay. Shown is the result of a 1% agarose-formaldehyde gel electrophoresis of wtCFTR-mRNA, blank and capsaicin-loaded nanocapsules either naked or surface-loaded with wtCFTR-mRNA at P/N charge ratio 75 in water and in transfection medium (Opti-MEM™) after 24 h incubation at 37 °C. Marker: RiboRuler™ High Range RNA Ladder (Thermo Fisher Scientific, Waltham, MA, USA).

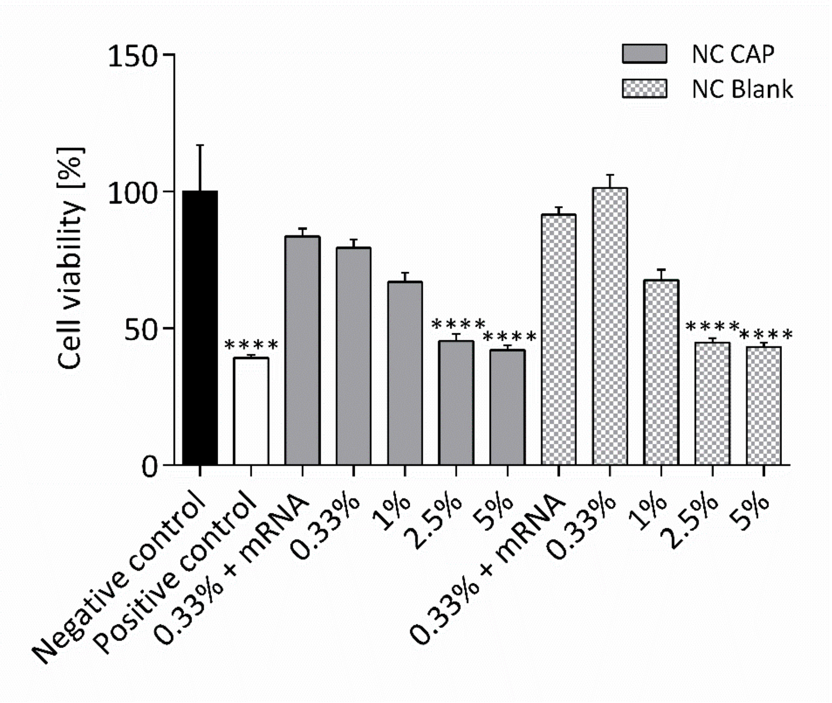

Figure S2. Effect of CS nanocapsules on the viability of 16HBE14o- cells. Cells were incubated with blank nanocapsules (NC Blank) and nanocapsules loaded with capsaicin (NC CAP) at varying concentrations in Opti-MEM™ for 24 h before an MTT assay was conducted. Concentrations correspond to 33 µM, 100 µM, 250 µM and 500 µM capsaicin in cells incubated with NC CAP. Cell culture medium was used as negative control; Triton® X-100 was used as positive control. Significance was calculated compared to the negative control ($p \leq 0.0001$ (****); n=3).
